# Biomedical application of TiO_2_NPs can cause arterial thrombotic risks through triggering procoagulant activity, activation and aggregation of platelets

**DOI:** 10.1007/s10565-024-09908-y

**Published:** 2024-08-07

**Authors:** Yiying Bian, Qiushuo Jin, Jinrui He, Thien Ngo, Ok-Nam Bae, Liguo Xing, Jingbo Pi, Han Young Chung, Yuanyuan Xu

**Affiliations:** 1 Key Laboratory of Environmental Stress and Chronic Disease Control & Prevention Ministry of Education, China Medical University, Shenyang, China; 2 Key Laboratory of Liaoning Province On Toxic and Biological Effects of Arsenic, China Medical University, Shenyang, China; 3 Program of Environmental Toxicology, School of Public Health, China Medical University. No, 77 Puhe Road, Shenyang North New Area, Shenyang, Liaoning 110122 People’s Republic of China; 4 College of Pharmacy, Seoul National University, Seoul, 151-742 South Korea; 5 Faculty of Pharmacy, Thai Binh University of Medicine and Pharmacy, Thai Binh City, 410000 Vietnam; 6 College of Pharmacy, Hanyang University, Ansan, Gyeonggido 426-791 South Korea; 7 Safety Evaluation Center of Shenyang Research Institute of Chemical Industry Ltd, Shenyang, 110021 China; 8 Center for Food and Bioconvergence, Seoul National University, Seoul, 08826 South Korea; 9 Group of Chronic Disease and Environmental Genomics, School of Public Health, China Medical University. No, 77 Puhe Road, Shenyang North New Area, Shenyang, Liaoning 110122 People’s Republic of China

**Keywords:** Titanium dioxide nanoparticles (TiO_2_NPs), Platelet (PLT), Calcium, Arterial thrombosis (AT)

## Abstract

**Background:**

Titanium dioxide nanoparticles (TiO_2_NPs) are widely used in medical application. However, the relevant health risk has not been completely assessed, the potential of inducing arterial thrombosis (AT) in particular.

**Methods:**

Alterations in platelet function and susceptibility to arterial thrombosis induced by TiO_2_NPs were examined using peripheral blood samples from healthy adult males and an in vivo mouse model, respectively.

**Results:**

Here, using human platelets (hPLTs) freshly isolated from health volunteers, we demonstrated TiO_2_NP treatment triggered the procoagulant activity of hPLTs through phosphatidylserine exposure and microvesicles generation. In addition, TiO_2_NP treatment increased the levels of glycoprotein IIb/IIIa and P-selectin leading to aggregation and activation of hPLTs, which were exacerbated by providing physiology-mimicking conditions, including introduction of thrombin, collagen, and high shear stress. Interestingly, intracellular calcium levels in hPLTs were increased upon TiO_2_NP treatment, which were crucial in TiO_2_NP-induced hPLT procoagulant activity, activation and aggregation. Moreover, using mice in vivo models, we further confirmed that TiO_2_NP treatment a reduction in mouse platelet (mPLT) counts, disrupted blood flow, and exacerbated carotid arterial thrombosis with enhanced deposition of mPLT.

**Conclusions:**

Together, our study provides evidence for an ignored health risk caused by TiO_2_NPs, specifically TiO_2_NP treatment augments procoagulant activity, activation and aggregation of PLTs via calcium-dependent mechanism and thus increases the risk of AT.

**Graphical Abstract:**

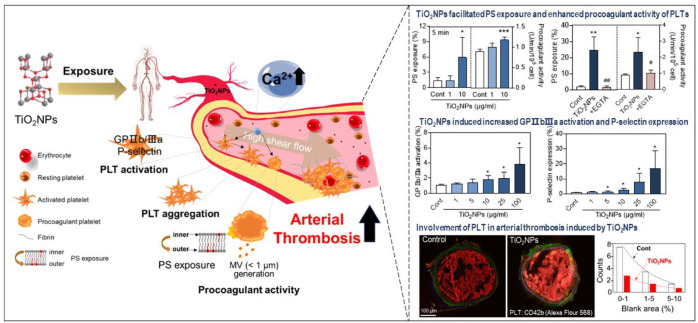

## Introduction

The titanium dioxide nanoparticles (TiO_2_NPs) that are smaller than 100 nm are widely utilized in biomedical fields such as photoimaging, drug delivery and biological analysis due to their antimicrobial properties, photocatalytic activity, excellent biocompatibility, and corrosion resistance(Najahi-Missaoui et al. 2020; Zhao and Castranova 2011). Often, intravenous injection is the norm, allowing the distribution of these nanoparticles to each organ via the bloodstream for therapeutic purposes. However, this process frequently leads to unexpected organ toxicity, including liver, kidney, and brain injuries(Shi et al. 2013). Given the significance of blood cells as the primary and essential contact component in the blood circulation process, it is noteworthy that toxicological research on these cells remains relatively scarce. Hence, the potential health risk of TiO_2_NP treatment, particularly from a perspective focusing on blood cells, is becoming a large concern(Lee et al. 2019; Naserzadeh et al. 2018; Qi et al. 2022; Shakeel et al. 2016).

The thrombosis, which is responsible for one in four deaths(2014), can be primarily categorized into venous, arterial, and microvascular thrombosis(Brandtner et al. 2021; Petri 2020). Our previous study have demonstrated that TiO_2_NPs have the ability to initiate procoagulant activity in red blood cells, ultimately culminating in the development of venous thrombosis(Bian et al. 2021). Although some reports have proposed that TiO_2_NPs can target platelets and cause microclots in microcirculation, their effects appear to be primarily restricted to platelet aggregation in vitro(Haberl et al. 2015). Nonetheless, in-depth thorough investigation of the underlying mechanism and the presence of other forms of platelet dysfunction still remain unexplored.

Recently, the activation and aggregation of platelets, as indicated by an elevated expression of the integrin αIIbβ3 (GPIIb/IIIa complex, CD41/CD61) and the transmembrane protein P-selectin within the alpha granules, have been strongly linked to thrombotic disorders(Huang et al. 2019; Qiao et al. 2018; Yeini and Satchi-Fainaro 2022). In addition, recent studies have indicated that the pro-coagulant activity of platelets (PLTs) plays a crucial role in the development of thrombosis, which is primarily attributed to the level of phosphatidylserine (PS) exposed on the outer membrane surface and the release of microvesicles (MVs)(Pang et al. 2018; Zlamal et al. 2023). The two processes are caused by an elevated calcium level, leading to hemostasis and thrombosis(Obydennyy et al. 2016; Varga-Szabo et al. 2009), ultimately pointing to the same outcome: the arterial thrombosis.

In this study, we initially established that intravenous administration of TiO_2_NPs swiftly instigates procoagulant activity within platelets by enhancing phosphatidylserine exposure and microvesicle production. Additionally, we observed prompt platelet activation and aggregation following TiO_2_NP treatment through the activation of GPIIb/IIIa and the expression of P-selectin. These findings indicate that TiO_2_NPs possess the potential to alter platelet function and promote the carotid artery thrombosis, a process that necessitates an elevated intracellular calcium level. Indeed, we also observed dysregulation of blood flow signals and arterial thrombosis along with increased PLT deposition using a carotid artery thrombosis mice model. These results provide clear evidence for the risk of PLT-related arterial thrombosis caused by TiO_2_NP treatment, and help to improve the understanding of the arterial thrombotic risk caused by TiO_2_NPs.

## Methods

### Materials

TiO_2_NPs (anatase, nanopowder, < 100 nm particle size), trisodium citrate, HEPES, prostaglandin E1 (PGE1), glutaraldehyde, EDTA, EGTA, ferric chloride, urethane, clopidogrel, and bovine serum albumin were obtained from Sigma-Aldrich (St. Louis, MO, USA). Thrombin and collagen were obtained from Calbiochem (San Diego, CA, USA) and Chrono-log (Havertown, PA, USA), respectively. Fluorescein isothiocyanate (FITC)-labeled anti-CD62P antibody (anti-CD62P-FITC Ab), FITC-labeled PAC-1 (PAC-1-FITC), and FITC-labeled annexin V (annexin V-FITC) were from BD Biosciences (San Jose, CA, USA). Fluo-4 acetoxymethyl ester (Fluo-4 AM) was obtained from Invitrogen (Carlsbad, CA, USA).

### Blood collection and preparation of human platelets (hPLTs)

This work was supported by the Ethics Committee of the Health Service Center at Seoul National University with the approval from the Institutional Review Board of Seoul National University (IRB No. 1702/003–004, 5, March, 2019). To simplify our study design, we selected only healthy male donors aged 20–30 years, excluding risk factors for thrombosis such as gender, age, and disease, and taking any medication in the past 2 weeks. For platelet-rich-plasma (PRP) preparation, whole blood with 3.2% trisodium citrate was centrifuged at 150 × *g* for 15 min and platelet cell count in PRP was adjusted 3 × 10^8^ cells/mL by diluting with platelet-poor-plasma (PPP) on the day of experiments.

### *Characterization of TiO*_*2*_*NPs*

TiO_2_NPs were not surface treated and insoluble in water, hydrochloric acid, or nitric acid. The particles were dispersed in distilled water and sonication was performed before each experiment, using an ultrasonicator with a maximum output of 150–200 W for 15 s to prevent agglomeration. TiO_2_NPs were dried and observed with scanning electron microscope (SEM) (SU8010, Hitachi Limited, Japan) to examine the size distribution. Detailed statistical analysis (Nano measure 1.2 and OraginPro2021) of TiO_2_NPs were performed by random measurement of 100 nanoparticles in the images taken by SEM. The dynamic particle size of the nanoparticles was evaluated using a Malvern laser particle size analyzer (DLS-7000, Otsuka Electronics, Co., Osaka, Japan). The sample was weighed and dispersed in deionized water (0.1% mass fraction), sonicated for 3 min, adjusted to pH 7.4 with NaOH or HCl. The zeta potential was measured with a nanoparticle size analyzer (ZS-90, Malvern Instruments, UK). All data were repeated three times and averaged. Technical support was provided by Beijing Standard Spectrum Testing Technology Co.

### Observation of cellular uptake of TiO_2_NPs under transmission *electron* microscope (TEM)

Cellular uptake of TiO_2_NPs by PLTs was observed using TEM following these procedures. After incubating isolated PLTs for 24 h with distilled water (as a control) and 25 µg/mL of TiO_2_NPs dispersed in distilled water as a colloidal suspension, cell fixation was carried out using a 2% glutaraldehyde solution in the refrigerator for 1 h, followed by post-fixation using 1% osmium tetroxide for 2 h. Subsequently, en-bloc staining was done with 0.5% uranyl acetate for 30 min, followed by serial dehydration steps with 30, 50, 70, 80, 90% ethanol (1 time each) and 100% ethanol (3 times). Transition and infiltration were then gradually performed using propylene oxide (10 min, twice), once with propylene oxide and Spurr's resin (1:1) for 2 h, and finally with Spurr's resin in a desiccator overnight. The next day, infiltration was completed with fresh Spurr's resin for 2 h in the desiccator, and samples were then kept in a 70 °C oven overnight for polymerization. Finally, samples were examined under TEM (JEOL, JEM 1010).

### Measurement of PLT aggregation

After incubation with TiO_2_NPs (0.5, 1, 5, 10, 25, and 100 μg/ml; 5 min, 30 min, and 60 min) at 37 °C, the number of individual PLTs per microliter was calculated using optical microscopy and the degree of PLT aggregation was assessed based on the count of single cells. Data were presented as percentages of PLT aggregation. Besides, PLT aggregation induced by TiO_2_NP treatment under physiology-mimicking conditions was observed by the above method under stimulation with thrombin (0.6–0.8 U/ml), collagen (2–4 μg/ml). PLT suspension was exposed to a shear rate of 1500 s^−1^ for 10 min at 37 °C using a programmable cone-plate viscometer. The shear-stressed platelets were fixed with 0.5% glutaraldehyde in Tyrode's buffer (134 mM NaCl, 2.9 mM KCl, 1.0 mM MgCl_2_, 10.0 mM HEPES, 5.0 mM glucose, 12.0 mM NaHCO_3_, 0.34 mM Na_2_HPO_4_, and 0.3% bovine serum albumin, pH 7.4) to evaluate platelet aggregation. The number of single platelets per microliter was counted under a phase-contrast light microscope. Prior to counting, platelet suspensions were diluted to approximately 300 to 500 particles in 5/25 squares. The variations between two different fixed samples from the same platelet suspension were typically less than 3% from the mean. Optical observation and counting methods can effectively exclude the potential influence of nanomaterial agglutination on platelet aggregation, ensuring accurate results.

### Evaluation of LDH leakage

Lactate dehydrogenase (LDH) leakage from PLT was measured by spectrophotometry. After incubation with TiO_2_NPs for 5 min, the supernatant obtained from the centrifuge reaction mixture was used for LDH determination (digitonin 50 μM treatment for 1 h was used as positive control). The degree of cell lysis was expressed as a percentage of total enzyme activity compared to control incubation with cleavage with digitonin.

### Flow cytometry analysis

The PLTs were obtained by centrifugation as described above. After PLTs were exposed with TiO_2_NPs at 37 °C for 10 min, the level of P-selectin and the activation of glycoprotein GPIIb/IIIa were determined by staining with CD62P-FITC and PAC-1-FITC for 20 min, respectively. PS exposure and MV generation in PLTs were examined by staining with both annexin V-FITC and anti-CD42b-PE Ab (as PLT identifier). MVs are described as cells with a diameter of less than 1 µm. Therefore, the cell population of MVs was differentiated by flow cytometry SSC versus FSC, while MV production in PLTs was confirmed by the PLT surface marker CD42b. Intracellular calcium level was determined by pre-loading fluo-4/AM (5 µM) for 45 min. All the determinations were conducted using FACS Calibur (Becton Dickinson, USA), and Cell Quest Pro software was used to collect and analyze data from 10,000 events. When analyzing, draw the part of annexin V-FITC positive staining and CD42b positive staining in the FACS program, and then export the percentage. Upon analyzing, for example, the PS exposure on platelets, we identified the portion of annexin V-FITC positive staining and CD42b positive staining in the FACS program. Subsequently, we exported the corresponding percentage.

### A prothrombinase assay

The prothrombinase assay was applied to assess procoagulant activity. Specifically, TiO_2_NP-treated PLTs were further induced into thrombin generation by adding 5 nM Xa factor, 10 nM Va factor and 2 μM prothrombin. Then, chromogenic substrate S2238 (Chromogenic, Milan, Italy) was used to measure the generated thrombin after adding a stop buffer (50 mM Tris–HCl, 120 mM NaCl, 2 mM EDTA, pH 7.9). Calculation of thrombin production rate was based on the absorbance change at 405 nm from the calibration curve.

### Animals

The experimental C57BL/6 J mice (male, 12 weeks old) were kept in a clean environment without specific pathogens and fed with SPF chow diet and distilled water. The indoor temperature was controlled at 23 ± 1 ℃, the humidity was 55–70%, and the circadian rhythm was alternated (12 h/12 h). Mice were randomly divided into two groups: control group and exposed group (TiO_2_NPs, 25 mg/kg, intravenous injection). Mice were anesthetized using 3–5% isoflurane by inhalation, and blood samples and other relevant indicators were collected from the mice under anesthesia with a mercy endpoint. All animal experiments were reviewed and approved by the Animal Ethics Committee of China Medical University (CMU20231000). For this specific experiment, we strictly adhered to the triple-blind methodology, ensuring that neither the experimental observer, the research subject, nor the data analyst was aware of the experimental specifics until the official release of the analysis results. Furthermore, taking into account the blinding techniques employed in animal studies, mice were subsequently randomized and assigned to either the control or exposure groups (TiO_2_NPs, 25 mg/kg, intravenous administration).

### *Ex vivo *assessment using blood cell analyzer

Blood (30 μl) was collected from mice tail with EDTA-K_2 -_containing tube 1 h post TiO_2_NP injection. Then fresh blood samples were tested by an automated five-classification animal blood cell analyzer (IDEXX ProCyte Dx, Japan) for the following indicators: PLT count (performed by both impedance (PLT-I) and optical (PLT-O) method), percentage of PLT-larger cell ratio (L-PCR%), PLT crit (PLT%), mean PLT volume (MPV), mean PLT volume/PLT count (MPV/P), PLT distribution width (PDW), red blood cell count, percentage of hematocrit, hemoglobin, and white blood cell-related indexes.

Ex vivo measurement of mPLT aggregation was conducted by an animal blood cell analyzer with impedance method, which only allows one single cell go through. The PLT aggregation rate was calculated by the formula: (PLT_Cont_-PLT_TiO2NPs_)/PLT_Cont_ × 100)%.

### Arterial thrombosis in mice

*Arterial thrombosis mouse model:* The left common carotid artery was isolated from the left side of the trachea using 3 × 5 mm tin foil to isolate the surrounding tissues. Afterwards, 1 × 2 mm filter paper was fully submerged with 5% FeCl_3_ and applied to the left common carotid artery for 15 min.

*Ultrasound observation:* The blood flow signal by the Doppler ultrasound in the mouse model was slightly adjusted based on previous study(Jing et al. 2023; Wang and Xu 2005). Mice were anesthetized with isoflurane inhalation at 3% and maintained at 2% throughout the procedure and then cleaned of hair on the neck and chest to facilitate ultrasound. AVINNO6 LAB small animal Doppler ultrasound system (VINNO Co., China) was used. The left common carotid artery was imaged and the flow signals (including velocity, flow volume, internal diameter, perfusion index, heart rate) were quantified.

### Thrombuspath*o*logical experiment

The tissue of the common carotid artery was collected for the pathological experiment. The thrombus tissue was embedded using the frozen section embedding agent Sakura OCT (a water-soluble mixture of polyethylene glycol and polyvinyl alcohol), sectioned at 6 μm thickness with frozen sectioning machine (CM 1950, LEICA, Germany), and collected using adhesive slides to observe thrombus morphology under a general light microscope. By measuring the percentage of each blank area in the thrombus (the area that blood flow can pass through), it is artificially divided into the following groups: 0–1, 1–5, and 5–10% of the total area. Less blank area means more compact thrombus.

Immunofluorescence observations for frozen sections were examined by adding α-fibronogen with Alexa Fluor® 488 and CD42b with Alexa Fluor® 568, respectively. Then, the samples were sealed with a blocking buffer containing a liquid with an anti-fluorescent cracking agent.

### Statistical analysis

All data are presented as the mean and standard deviation. Data were subjected to Student's *t*-test or two-way ANOVA followed by Duncan's multiple range test. In all cases, a *P* value < 0.05 was considered statistically significant. The asterisk represents significant differences from the control group (***P < 0.001; **P < 0.01; *P < 0.05). The pound represents significant differences from the TiO_2_NP treatment group (^###^*P* < 0.001; ^##^*P* < 0.01; ^#^*P* < 0.05).

## Results

### *Characterization of TiO*_*2*_*NPs and the uptake by human platelets (hPLTs)*

**Fig. 1:**
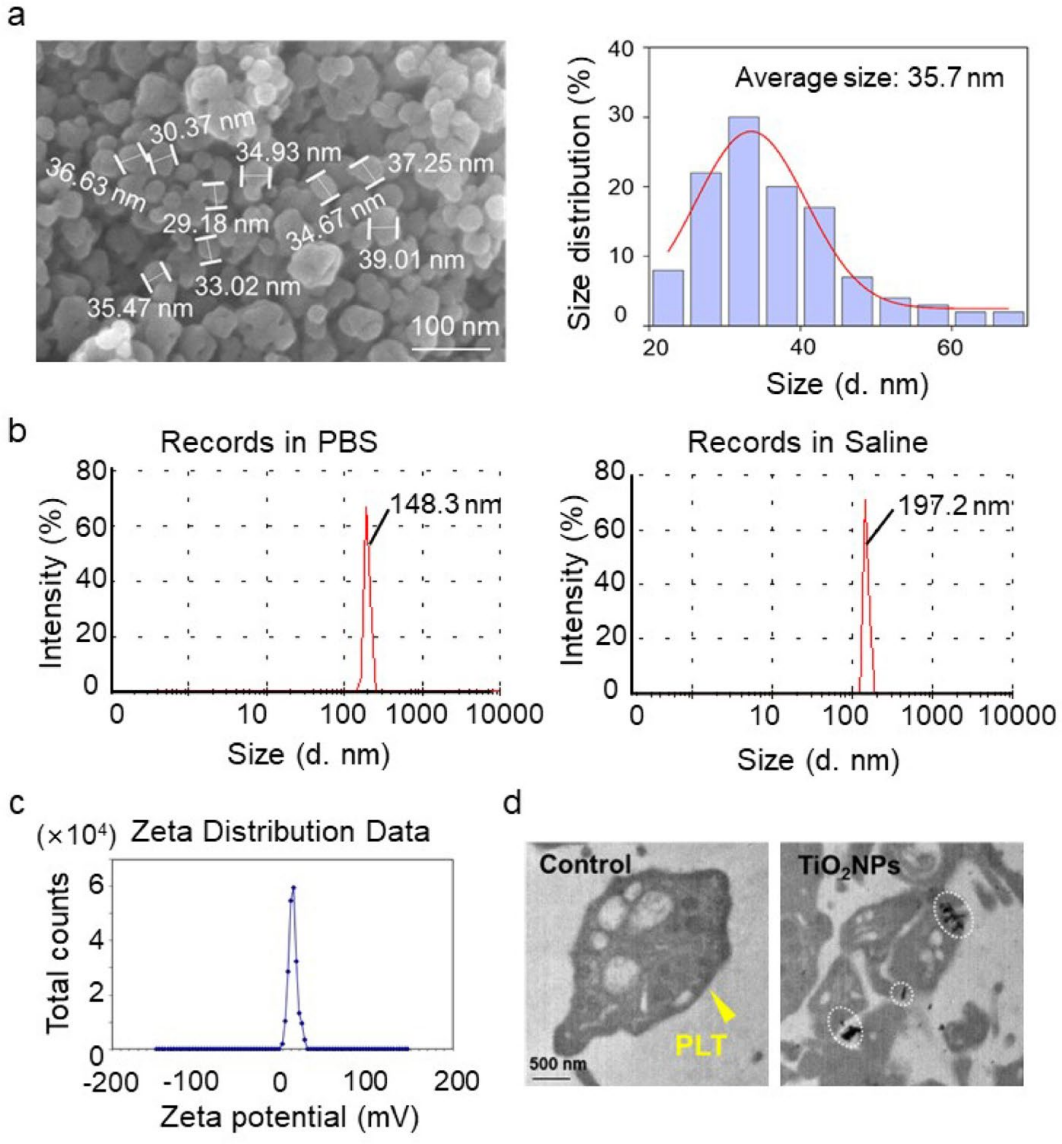
Characterization of titanium dioxide nanoparticles (TiO_2_NPs) and the uptake by human platelets (hPLTs). (**a**) SEM observation showed the size of TiO_2_NPs between 20 and 70 nm with an average diameter of 35.7 nm. Scale bar: 100 nm. (**b**) The dynamic size distribution of TiO_2_NPs by the intensity in saline and in PBS with a peak of distribution at 148.3 nm and 197.2 nm, respectively. (**c**) The zeta potential of TiO_2_NPs in distilled water was + 11.7 mV as tested by a nanoparticle size analyzer. (**d**) TEM observation of uptake of control (distilled water) and TiO_2_NPs by hPLTs. Yellow arrowhead: hPLT; black scale bar: 500 nm; dashed circles: TiO_2_NPs within hPLTs; dashed box: TiO_2_NPs adhered to hPLT membrane surface

The physicochemical characterization of TiO_2_NPs was examined by SEM and DLS. The size distribution of TiO_2_NPs was in the range of 20 to 70 nm with an average diameter of 35.7 nm (Fig. 1a), which was randomly calculated from 100 particles shown in SEM images. DLS data further showed that the average size by intensity was 148.3 nm in saline (with 10% FBS) and 197.2 nm in PBS, respectively (Fig. 1b). The zeta potential of TiO_2_NPs was + 11.7 mV at pH 7.4 (Fig. 1c). Interestingly, TEM observation showed that TiO_2_NPs were within hPLTs (dashed circles) or adhered to hPLT membrane (dashed box) (Fig. 1d), indicating altered consequences linked to PLT functions in response to TiO_2_NP treatment.

### *TiO*_*2*_*NPs enhance hPLT procoagulant activity through PS exposure and MVs generation*

**Fig. 2:**
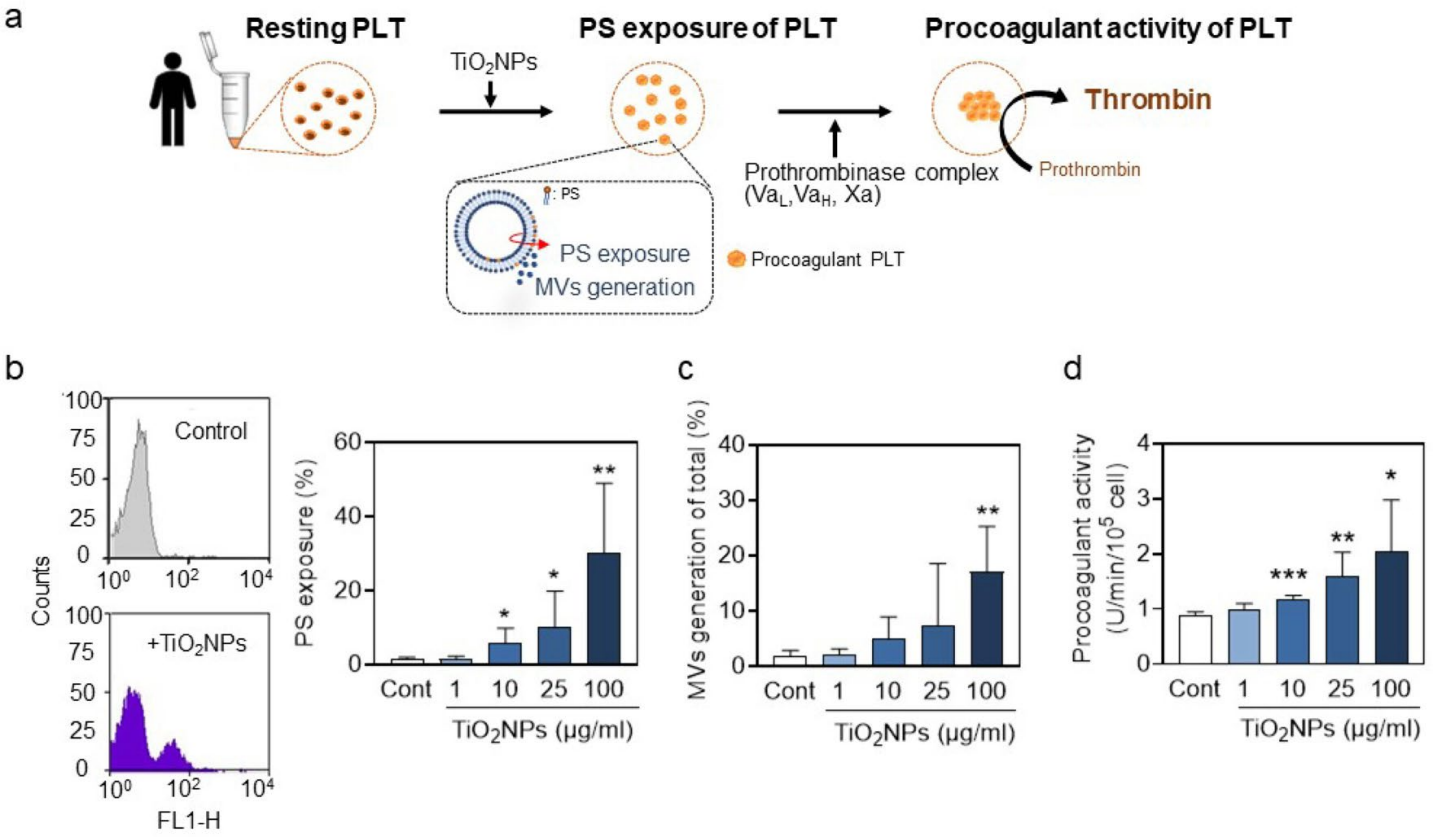
TiO_2_NPs enhance hPLT procoagulant activity through phosphatidylserine (PS) exposure. (**a**) A diagram of prothrombinase assay showing PS exposure of PLTs in the process of thrombin generation indicating increased procoagulant activity after TiO_2_NP treatment. (b-c) Isolated hPLTs were treated with various concentrations (1, 10, 25, and 100 μg/mL) of TiO_2_NPs at 37 °C for 10 min. Both (**b**) PS exposure and (**c**) MV generation were measured by flow cytometry. (**d**) The procoagulant activity was determined by thrombin generation using prothrombinase assays as mentioned in Methods. Values are mean ± SD of the independent experiments from different blood donors (n = 5–7). The asterisk represents significant differences from the control group (***P < 0.001; **P < 0.01; *P < 0.05)

In resting PLTs, PS are commonly in the inner leaflet of the membrane. Upon stimuli, PS are externalized to the outer leaflets and microvesicles (MVs, < 1 μm) are released, both of which participate in coagulation pathway and then promote the production of thrombin from prothrombin under prothrombinase complex (Va, Xa)(Lentz 2003). To estimate procoagulant activity of PLTs induced by TiO_2_NPs, we treated hPLTs with TiO_2_NPs for 10 min, determined PS exposure and MVs generation using FACs, and estimated thrombin generation under prothrombinase complex (Va, Xa) (Fig. 2a). PS exposure and MVs were significantly increased in a concentration-dependent manner after TiO_2_NP treatment (Fig. 2b and 2c). In parallel, with a prothrombinase assay, TiO_2_NP treatment accelerated thrombin generation in hPLTs, reflecting increased procoagulant activity (Fig. 2d).

### *TiO*_*2*_*NPs induce hPLT aggregation and activation *via* GPIIb/IIIa activation and increased P-selectin level*

**Fig. 3:**
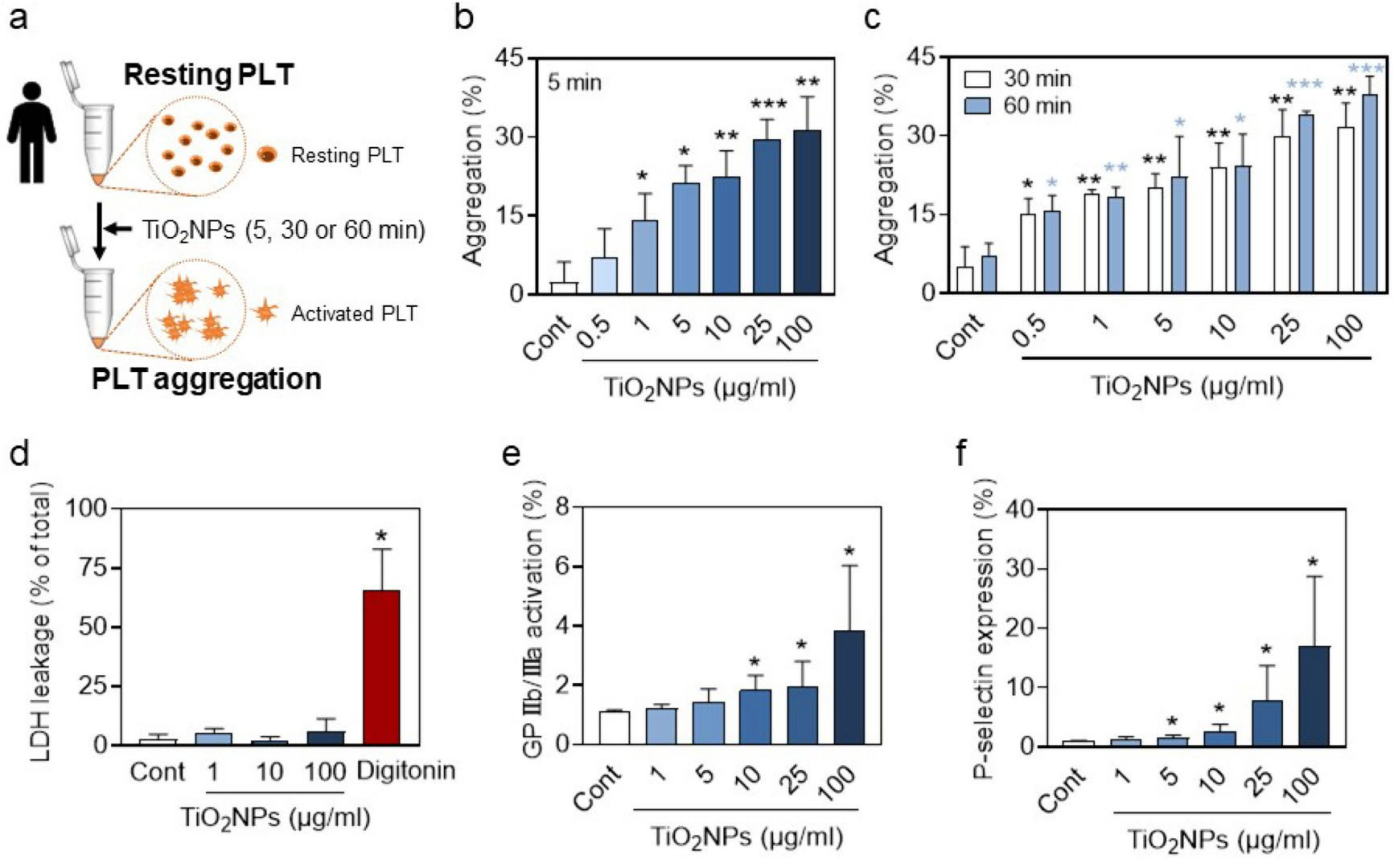
**T**iO_2_NPs induce hPLT aggregation and activation through increased GPIIb/IIIa activation and P-selectin level. (**a**) In vitro experimental method using PLTs freshly isolated from health volunteers. (b-c) Percentage of hPLT aggregation after hPLTs were treated to various concentrations (0.5, 1, 5, 10, 25, and 100 μg/ml) of TiO_2_NPs for (**b**) 5 min, (**c**) 30 min and 60 min. (**d**) LDH leakage of hPLTs induced by TiO_2_NPs. DIG, digitonin 50 μM treatment for 1 h, used as positive control. (**e**) GPIIb/IIIa activation and (**f**) P-selectin level were measured by flow cytometry. Values are mean ± SD of the independent experiments from different blood donors (n = 3–5). The asterisk represents significant differences from the control group (***P < 0.001; **P < 0.01; *P < 0.05)

To exam whether TiO_2_NPs induce PLT aggregation, we used PRP freshly isolated from healthy volunteers and incubated hPLTs with TiO_2_NPs for 5 min, 30 min or 60 min as shown in Fig. 3a. In consequence, TiO_2_NPs caused PLT aggregation at 5 min in vitro (Fig. 3b). Upon 30 and 60 min exposure, TiO_2_NPs also caused PLT aggregation but did not show obvious time-dependent effects even when compared with 5 min exposure (Fig. 3c), indicating that TiO_2_NPs rapidly exaggerate PLT aggregation which occurs within 5 min. Next, we examined the leakage of LDH from hPLTs and found that no cytotoxicity was induced by TiO_2_NP treatment (digitonin was used as a positive control) (Fig. 3d). Glycoprotein IIb/IIIa (GPIIb/IIIa) plays a key role in the maintenance of PLT aggregation(Aliotta et al. 2021). In addition, P-selectin is an adhesion molecule belonging to the selectin family expressed on PLTs, and activated PLTs express high levels of P-selectin(Yeini and Satchi-Fainaro 2022). As shown in Fig. 3e and 3f, GPIIb/IIIa activation and P-selectin expression were both significantly induced by TiO_2_NP treatment. Together with Fig. 2 b and d, all the markers suggesting procoagulant activity are matched well with Scientific and Standardization Committee of the ISTH defined procoagulant platelets (Josefsson et al. 2023).

### *TiO*_*2*_*NP-induced hPLT aggregation is exacerbated under physiology-mimicking conditions*

**Fig. 4:**
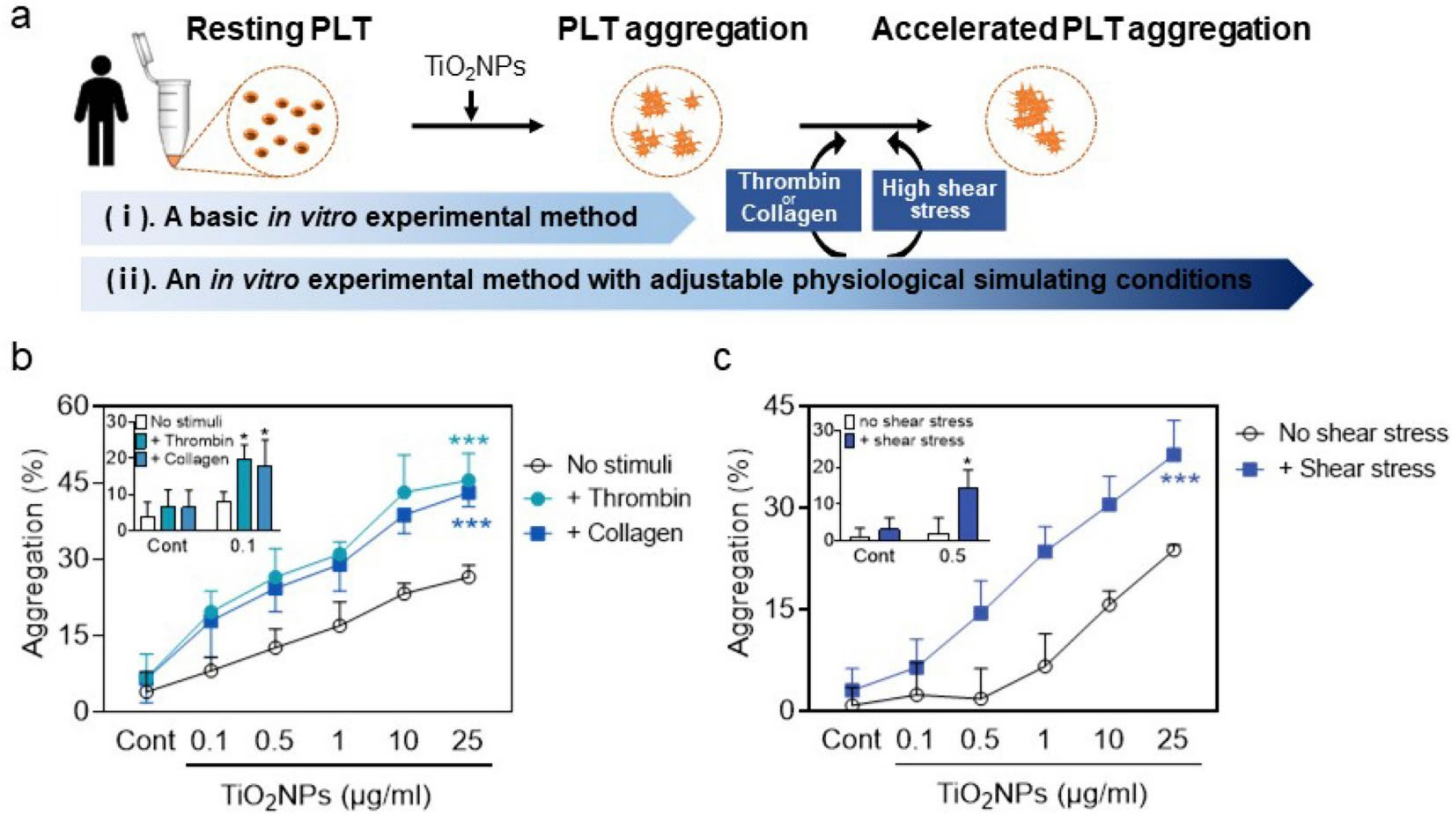
TiO_2_NP-induced hPLT aggregation is exacerbated under physiology-mimicking conditions. (**a**) A diagram showing a basic in vitro experimental method *versus* an in vitro experimental method with adjustable physiological simulating conditions. (**b-c**) Aggregation of hPLTs after adding thrombin (0.6–0.8 U/mL), collagen (2–4 μg/mL) or shear stress (1500 s.^−1^ for 3 min) under various concentrations (0.1, 0.5, 1, and 10 μg/ml) of TiO_2_NPs. Values are mean ± SD of the independent experiments from different blood donors (n = 3–5). The asterisk represents significant differences from the no stimuli or no shear stress group (***P < 0.001; *P < 0.05)

In the physiological system, the presence of physiological aggregators (e.g. thrombin and collagen) contributes to the heterogeneity in PLT responses (Aslam et al. 2013; Petzold et al. 2016; Sang et al. 2021). Additionally, vascular shear force plays an existential role in the physiological function of PLTs(Casa et al. 2015; Yagi et al. 2017). To assess the effect of TiO_2_NPs on PLT aggregation more accurately, we determined PLT aggregation using an in vitro experimental method with adjustable physiological simulating conditions (Fig. 4a, i) and compared the results with those from a basic in vitro experimental method (Fig. 4a, ii). After adding thrombin or collagen in vitro, accelerated TiO_2_NP-induced PLT aggregation was found (Fig. 4b). Similarly, after adding shear stress in vitro that simulates physiological high shear flow, enhanced PLT aggregation was observed (Fig. 4c). More significantly, the existence of physiological aggregators (thrombin and collagen) and high shear stress reduced the minimum toxic level of TiO_2_NPs for PLT aggregation from 1 μg/mL to 0.1 μg/mL (Fig. 4b, insert) and 0.5 μg/mL (Fig. 4c, insert), respectively, as compared to the basic condition. These data indicate that TiO_2_NPs boost more severe PLT aggregation under physiology-mimicking conditions.

### *Involvement of Ca*^*2*+^*in hPLT aggregation and procoagulant activity induced by TiO*_*2*_*NPs*

**Fig. 5:**
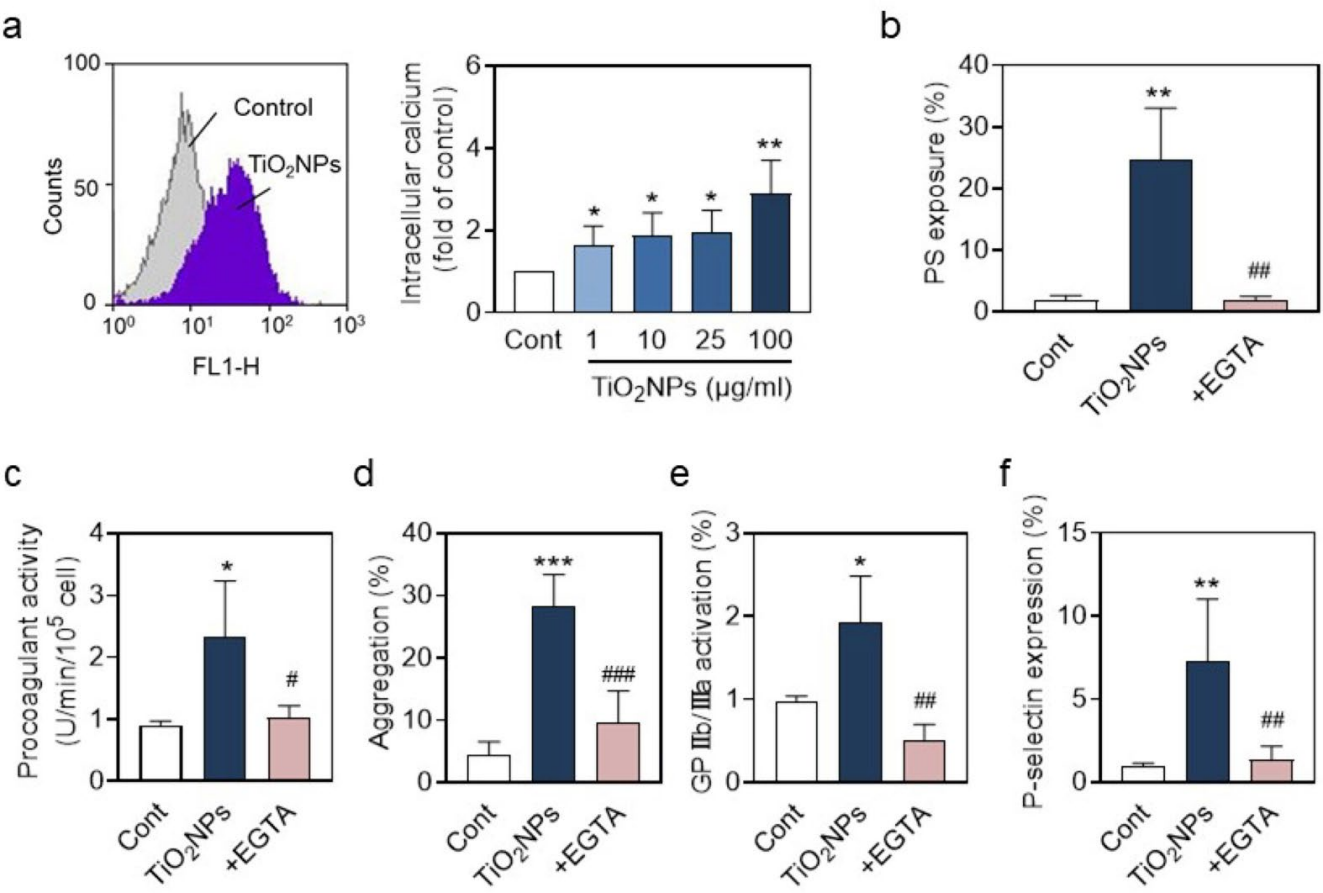
Involvement of Ca^2+^ in procoagulant activity, activation and aggregation of hPLT induced by TiO_2_NPs. (**a**) Intracellular Ca^2+^ level in hPLTs was measured by adding Fluo-4 AM after hPLTs were treated with 100 μg/mL TiO_2_NPs for 10 min. (**b**) PS exposure, (**c**) procoagulant activity, (**d**) hPLT aggregation, (**e**) GPIIb/IIIa activation, and (**f**) P-selectin level were performed by preloading a calcium chelating agent (EGTA) prior to TiO_2_NP treatment. Values are mean ± SD of the independent experiments from different blood donors (n = 3–6). The asterisk represents significant differences from the control group (***P < 0.001; **P < 0.01; *P < 0.05). The pound represents significant differences from the TiO_2_NP treatment group (^###^*P* < 0.001; ^##^*P* < 0.01; ^#^*P* < 0.05)

Intracellular Ca^2+^ plays a key role in PLT activation, aggregation and procogulant activity(Back et al. 2022; Xiang et al. 2021; Zhu et al. 2016). Here, we observed a concentration-dependent increase in intracellular Ca^2+^ level after TiO_2_NP treatment (Fig. 5a). To further assess the role of Ca^2+^ in PLT dysfunction, we treated hPLTs with EGTA (a chelator of Ca^2+^) prior to TiO_2_NP treatment. In consequence, increased PS exposure (Fig. 5b) and procoagulant activity (Fig. 5c) induced by TiO_2_NPs were both effectively reduced by EGTA. Concurrently, TiO_2_NP-induced hPLT aggregation was markedly blocked by EGTA (Fig. 5d). Meanwhile, elevated GPIIb/IIIa activation (Fig. 5e) and P-selectin level (Fig. 5f) by TiO_2_NP treatment were both significantly inhibited by EGTA.

### *TiO*_*2*_*NP treatment decreases PLT counts and increases large PLT ratio in mice*

**Fig. 6:**
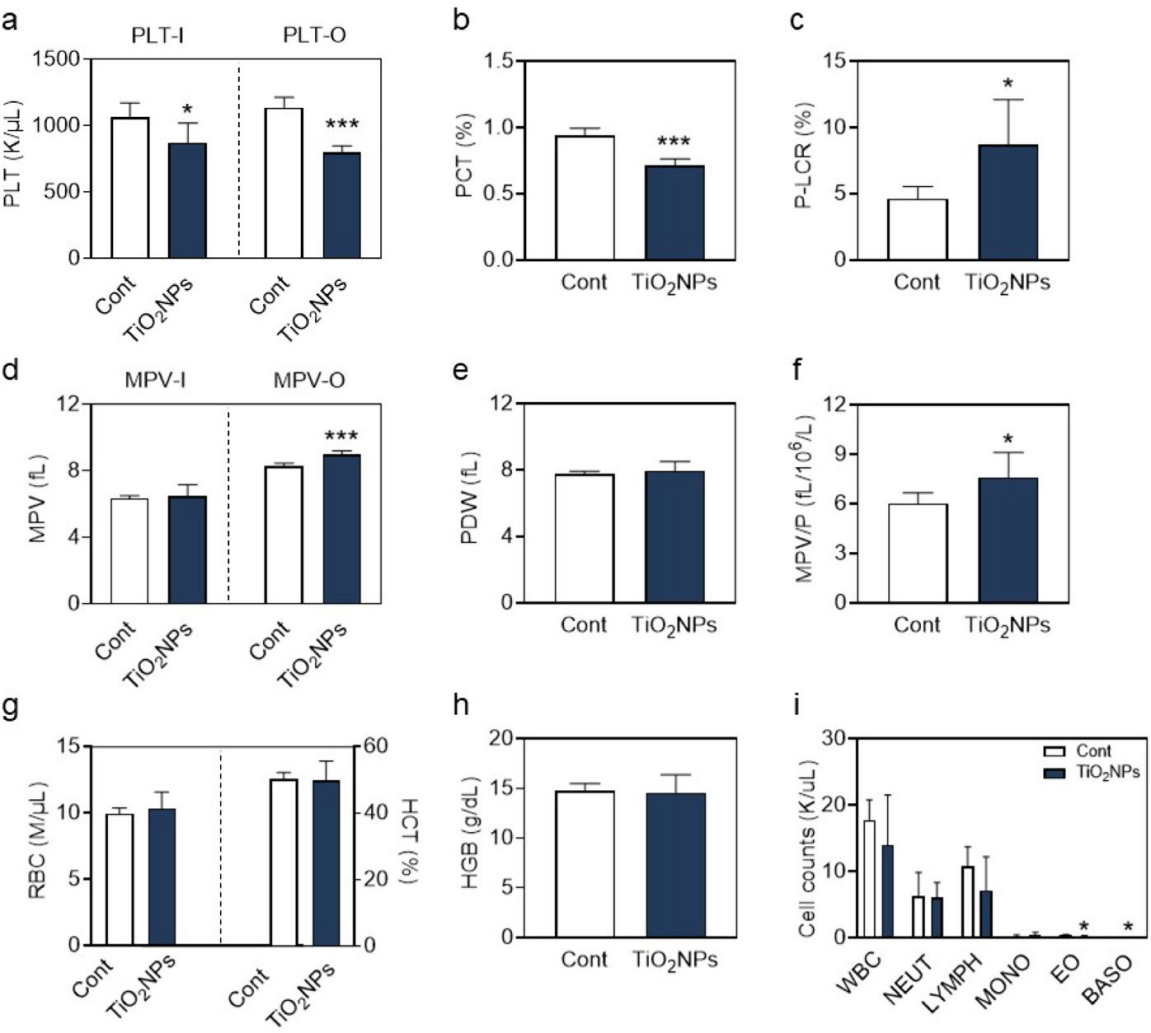
TiO_2_NP treatment results in decreased PLT counts and increased large PLT ratio in mice. TiO_2_NPs were intravenously injected into mice, and 1 h later, blood was collected from mice for hemocyte analysis. (**a**) PLT counts by impedance method (PLT-I), PLT counts by optical method (PLT-O), (**b**) percentage of plateletcrit (PCT%), (**c**) percentage of large PLTs (P-LCR%), (**d**) mean PLT volume by impedance (MPV-I) and by optical (MPV-O), (**e**) PLT volume distribution width (PDW), and (**f**) mean PLT volume/PLT count (MPV/P). At the same time, red blood cell-related indexes, such as (**g**) red blood cell count, percentage of hematocrit, (**h**) hemoglobin, and (**i**) white blood cell-related indexes, including white blood cell count and classification percentage were tested separately. Values are mean ± SD of the independent experiments from mice (n = 4–6). The asterisk represents significant differences from the control group (***P < 0.001; *P < 0.05)

To elucidate the in vivo effect of TiO_2_NPs, mice were intravenously injected with TiO_2_NPs and after 1 h, blood was collected and analyzed using a blood cell analyzer. It is clear from the data that PLT counts (Fig. 6a) detected by the impedance method (Fig. 6a, left) and the optical method (Fig. 6A, right), respectively, as well as PCT% (Fig. 6b), were significantly declined in TiO_2_NP-exposed mice. Meanwhile, P-LCR% was increased apparently in TiO_2_NP-exposed mice (Fig. 6c). In addition to P-LCR, MPV, PDW and MPV/P were all considered as potential indicators of PLT shape and function(Azab et al. 2011; Gasparyan et al. 2011; Han et al. 2013). Accordingly, increases in MPV (Fig. 6d) and MPV/P (Fig. 6f) after TiO_2_NP treatment were observed, but there was no change in PDW (Fig. 6e).

Moreover, TiO_2_NP-exposed mice showed no significant change in red blood cells (RBC) index including RBC counts (Fig. 6g, left), hematocrit (HCT%) (Fig. 6g, right), hemoglobin (HGB) (Fig. 6h), or white blood cells (WBC) index including neutrophils, lymphocytes, monocytes, eosinophils and basophils (Fig. 6i).

### *TiO*_*2*_*NPs trigger PLT dysfunction and locally alter arterial blood flow signals in mice*

**Fig. 7:**
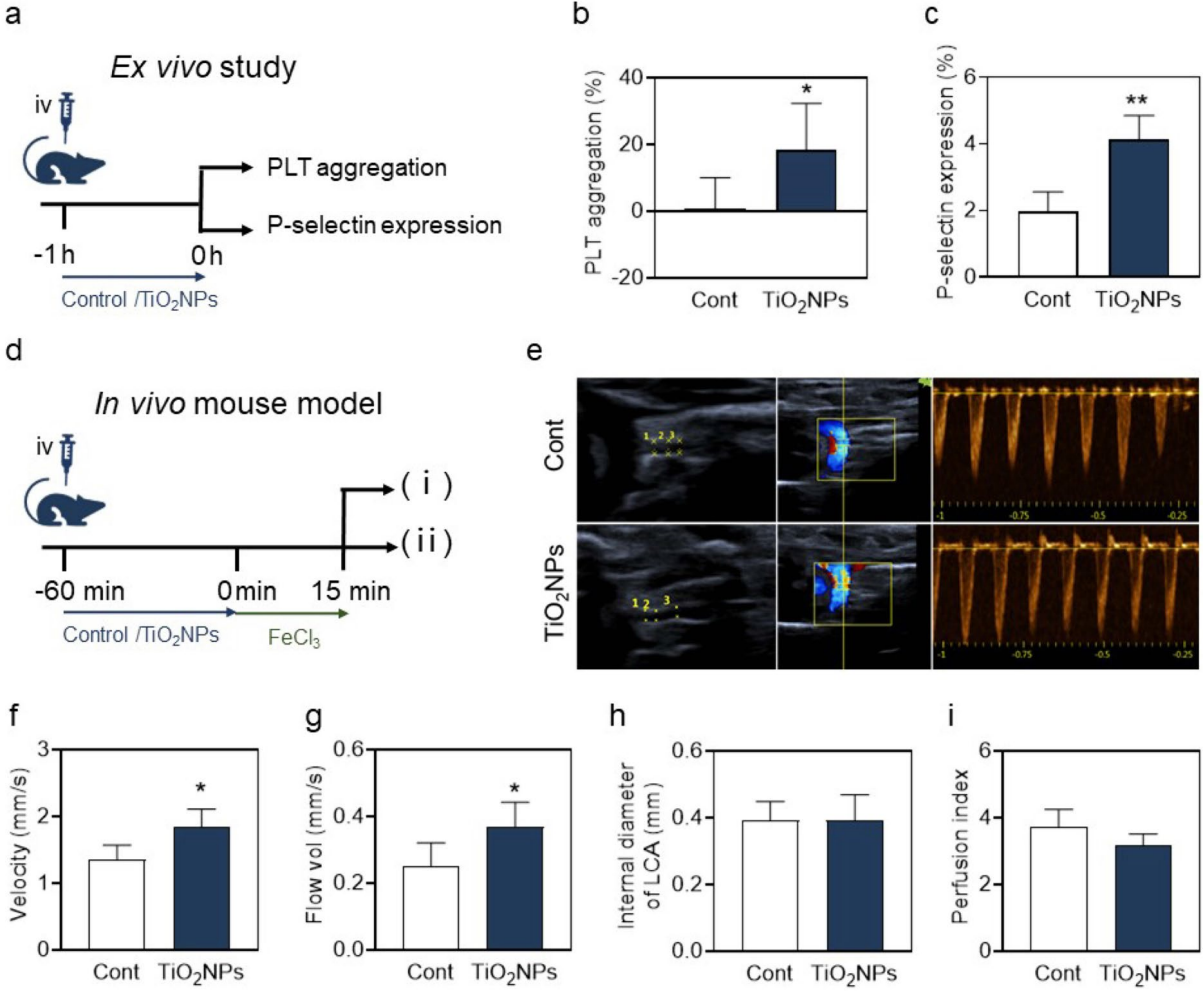
TiO_2_NPs trigger PLT dysfunction and locally accelerate arterial blood flow signals in mice. (**a-c**) A diagram of ex vivo study: 1 h after TiO_2_NP intravenous injection, (**b**) percentage of PLT aggregation was calculated by the impedance method and (**c**) P-selectin was measured using flow cytometry. (**d**) A diagram of the FeCl_3_-initiated AT mouse model. (**e-i**) Blood flow signals in the common carotid artery of mice were measured using small animal doppler color ultrasound. (**f**) Blood flow velocity, (**g**) blood flow volume, (**h**) internal diameter of the common carotid artery, and (**i**) vascular resistance index, were detected and quantified. Values are mean ± SD of the independent experiments from mice (n = 3–5). The asterisk represents significant differences from the control group (**P < 0.01; *P < 0.05)

To further determine whether TiO_2_NPs cause PLT aggregation and activation, we firstly did ex vivo study using PLTs isolated from mice (mPLTs) treated with TiO_2_NPs (Fig. 7a). TiO_2_NPs resulted in increased mPLT aggregation (Fig. 7b) and P-selectin (Fig. 7c) level 1 h post treatment. Numerous studies have shown that PLT activation and aggregation are closely related to thrombosis(Nayak et al. 2021; Yeung et al. 2018). Therefore, we established an AT-initiating mice model 1 h after TiO_2_NP treatment, and phenotypes were determined by color doppler ultrasonography (Fig. 7d, i). As shown in Fig. 7e, TiO_2_NP-exposed group showed different flow signals compared with control group. Specifically, TiO_2_NP-exposed mice represented obvious accelerated arterial blood flow velocity (Fig. 7f), increased blood flow volume (Fig. 7g), and a slightly declined trend in the vascular resistance index (Fig. 7i) with unconspicuous alterations in artery internal diameter (Fig. 7h). Such doppler data indicate thrombi ahead, which is consistent with early reports on the phenomena of increased blood flow signals and thrombus formation(He et al. 2023). Briefly, these all point to a critical clue that TiO_2_NP treatment increases the risk of AT.

### *TiO*_*2*_*NP treatment leads to carotid artery thrombosis with PLT deposition in mice*

**Fig. 8:**
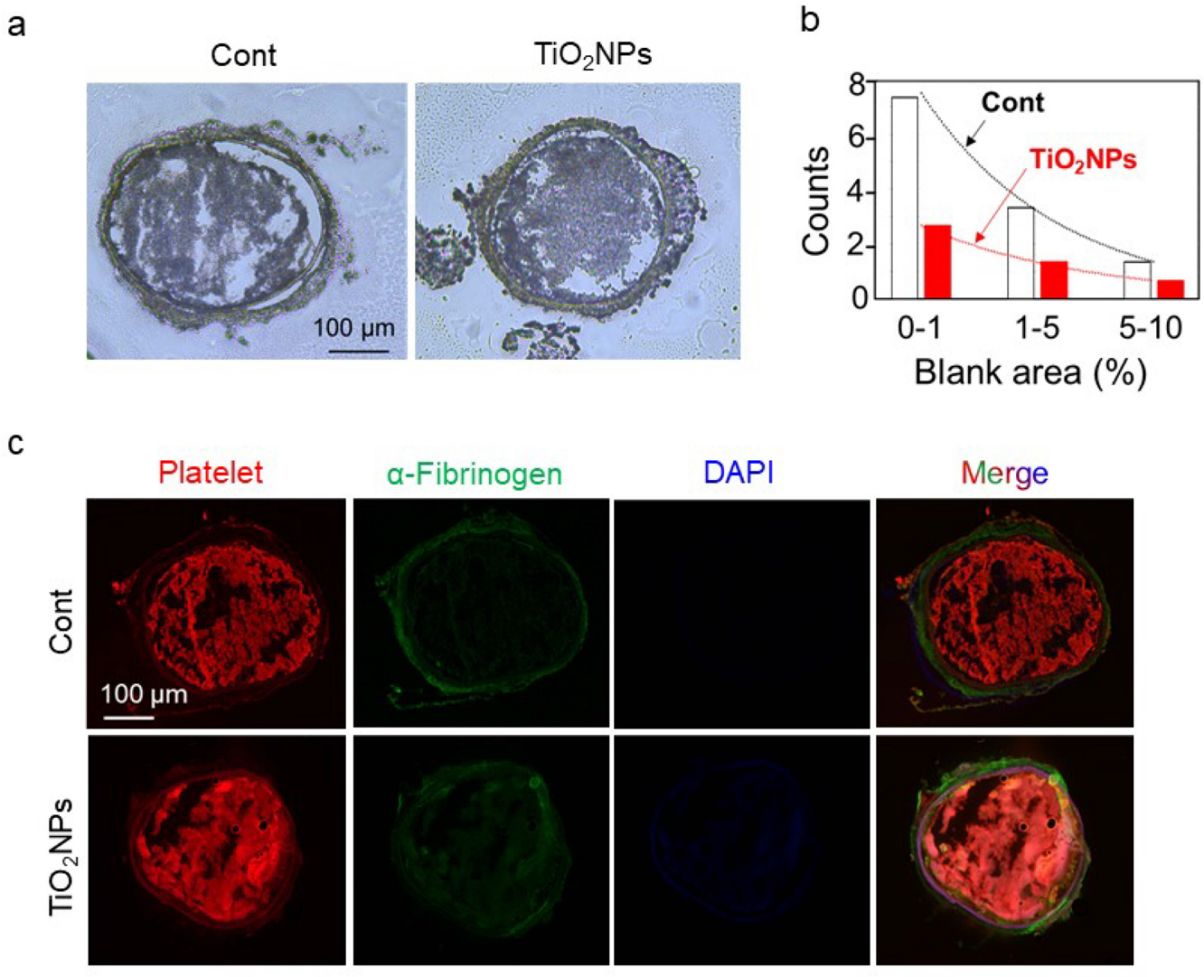
TiO_2_NP treatment leads to PLT-involved AT in mice. (**a**) Immunofluorescence staining of arterial thrombi was obtained using AT mouse model and (**b**) the blank area of aorta (area that blood flow can go through) was quantified as described in Methods. (**c**) The pathological composition of the thrombus was observed using immunofluorescence staining for PLTs (red, CD42b), α-fibrinogen (green) and DAPI (blue), respectively. Scale bar: 100 μm. Thrombus were obtained from independent mouse samples (n = 3)

In addition to the doppler ultrasonic detection, we conducted pathological sections of the arterial thrombus in mouse AT model (Fig. 7d, ii), and found increased thrombus formation in TiO_2_NP-exposed mice (Fig. 8a). Besides, we noticed that thrombi formed in TiO_2_NP-exposed mice were fuller and more compact, reflecting that the blood flow became more sluggish. Hence, we further analyzed the blank area of aorta (area that blood flow can go through) as mentioned in methods, to reflect the potential unobstructed capacity of blood flow. The total counts were separated into three distinguished levels including the tiny (less than 1% of the total aorta), the moderate (1–5% of the total aorta) and the loose blank area (5–10% of the total aorta). Consistent with the increase in thrombosis, mice exposed to TiO_2_NPs showed significantly less total blank areas or blank areas at each level compared to control mice, reflecting a decrease in blood flow patency in mice with AT induced by TiO_2_NPs (Fig. 8b). Finally, we stained the thrombi by immunofluorescence with CD42b (red color) to reflect the active role of PLTs in TiO_2_NP-induced AT. The red fluorescence indicating PLT deposition within the thrombi was increased in TiO_2_NP-exposed mice (Fig. 8c).

## Discussion

**Scheme 1:**
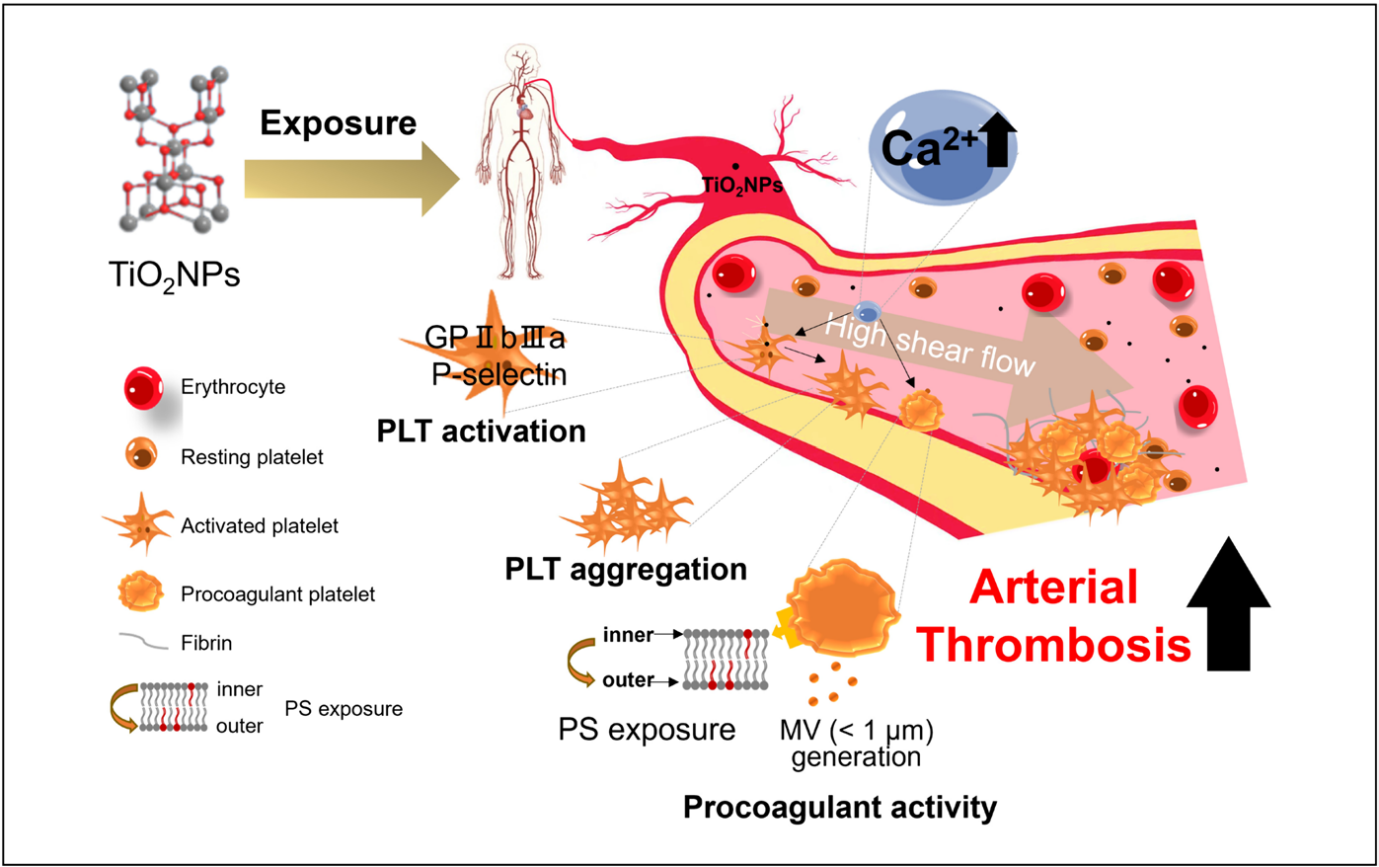
**S**uggested mechanism for the dysfunction of PLTs by TiO_2_NP. TiO_2_NPs can induce hPLT aggregation by elevating P-selectin, GPIIb/IIIa levels, and procoagulant activity through PS externalization and MV generation, involving intracellular calcium. PLT aggregation sensitization takes place within a physiology-mimicking system, thereby intensifying arterial thrombosis in mice via PLT deposition

The growing medical application of TiO_2_NPs has sparked critical consideration of their bio-safety. This study, for the first time, reveals that TiO_2_NPs can enhance procoagulant activity in isolated human platelets by increasing exposure of PS and the generation of MVs. Additionally, exposure to TiO_2_NPs rapidly triggers platelet activation and aggregation through increased expression of P-selectin and activation of GPIIb/IIIa. Here, intracellular calcium plays a role in both processes. Furthermore, TiO_2_NPs alter blood flow and exacerbate arterial thrombus formation in mice, accompanied by increased platelet deposition, underscoring the relevance of our findings to real in vivo conditions (Scheme 1).

Nanoparticles are often used intravenously in medical use, which inevitably have unintended side effects on blood cells. Numerous studies have shown that these particles can impact macrophages(Dey et al. 2021), neutrophils(Yamano et al. 2022), and lymphocytes. In addition, our previous research revealed that TiO_2_NPs can contribute to venous thrombosis by enhancing the procoagulant activity of red blood cells(Bian et al. 2021). In peripheral blood, platelets also play key roles in hemostasis and thrombosis(Koupenova et al. 2018). In this study, using isolated human samples, we discovered that platelets are more sensitive to TiO_2_NPs than red blood cells were in our previous finding. This heightened sensitivity was evident at concentrations as low as 1 μg/mL of TiO_2_NPs (after less than 10 min in vitro), which preceded the dysfunction of red blood cells, which occurred relatively high at 10 μg/mL after approximately 24 h in vitro. The findings of this study indicate that intravenous administration of TiO_2_NPs may pose a significant risk to blood cell function and homeostasis. It is crucial for future research to further investigate the potential long-term effects of these nanoparticles on blood cell function and cardiovascular health.

The reports on toxicity of NPs are always controversial due to the ability to release ionic form from metal NPs. Some studies claim that the toxicity of metal NPs is due to the metal ions released by NPs, while others argue that the toxicity of metal NPs is due to the small particle size of NPs themselves and other physicochemical properties as additional influencing factors(Bian et al. 2019; Ye et al. 2018; Zhang et al. 2020). For instance, zinc oxide and copper oxide nanoparticles (ZnONPs and CuONPs) are easy to release their ionic forms and their main toxicity is considered to be caused by the metal ions(Liu et al. 2016; Wang et al. 2016). However, with respect to silver nanoparticles (AgNPs), toxic effects are currently attributed into two aspects, the metal ion (Ag^+^) effect and the NP effect, respectively(Poynton et al. 2012). Most earlier studies suggest that it is Ag^+^ released from the AgNPs exerting a significant influence on protein regulation and the induction of cellular stress(Zhang et al. 2020). Different from this perspective, our previous study found that the level of Ag^+^ released from AgNPs as detected by ICP-MS method was negligible (< 0.01%) and hardly initiated a significant response of the cell(Bian et al. 2019), indicating that the effect of NPs themselves rather than the ion effect is the main contributor of AgNP toxicity. A recent study has revealed that though the ions of AgNPs cause a certain degree of toxicity, but not as severe as the toxicity due to their physical characteristics as NPs(Cvjetko et al. 2017). Unlike those typical metal NPs possessing the capability of releasing ionic forms, the level of metal titanium ions (Ti^4+^) dissociated from TiO_2_NPs is extremely low due to the special nature of titanium(Prokopiuk et al. 2023; Qin et al. 2017). Generally speaking, the toxic effects of TiO_2_NPs are proved to be mainly based on their unique NP properties.

TiO_2_NPs possess a range of physicochemical attributes, including diverse particle sizes, various crystalline forms (such as anatase and rutile), and distinct surface modifications (including surface charge and coatings). These characteristics play a pivotal role in determining the NPs' biological properties. Surface functionalization of TiO_2_NPs with negatively charged moieties has been demonstrated to mitigate the erythrocyte aggregation effects elicited by these NPs, potentially attributed to the formation of a complex system on the NP surface resulting from surface modification (Han et al. 2012). Notably, anatase TiO_2_NPs is renowned for its superior bioactivity compared to the rutile form, and in combination with smaller particle sizes, it may exacerbate toxicity (De Matteis et al. 2016; Shi et al. 2013), as evidenced by our prior investigation using human erythrocytes (Bian et al. 2021). Furthermore, previous studies have consistently reported that rutile-type TiO_2_NPs do not significantly affect PLT activation(Bihari et al. 2010). However, our novel research reveals that anatase-type TiO_2_NPs induce notable PLT activation, even at lower concentrations. Given these intriguing findings, we believe that further exploration of the thrombotic risks mediated by PLTs in future studies involving various TiO_2_NPs is imperative. Such investigations could provide valuable insights into the safety profile and potential therapeutic applications of these nanoparticles.

Physiological factors play important regulatory roles in the functional performance of PLTs(Sang et al. 2021). Flowing blood generates a frictional force called shear stress(Souilhol et al. 2020), and a common pathological symptom for myocardial infarction and ischemic stroke is thrombotic blood flow obstruction that forms at high shear rates in the arteries(Casa and Ku 2017). Thrombin, collagen, and ADP are currently considered to be the most potent physiological agonists of PLTs. In the present study, under physiology-mimicking conditions, we found that TiO_2_NP-induced hPLT aggregation was dramatically exacerbated. Although we tried our best to simulate in vivo system by adding physiological aggregators and imposing high shear stress, and as expected, observed increased PLT aggregation, the real human body is a large complex system quite difficult to completely and perfectly simulate.

Maintaining the intracellular concentration and distribution of free calcium ions in dynamic equilibrium is vital for cellular life activities. This balance is achieved through complex calcium ion permeation and transport mechanisms within the cell membrane and organelles. However, EGTA primarily chelates extracellular calcium, thus limiting its accuracy in assessing [Ca^2+^]_i_ levels. In past studies, MAPTAM (Korchak et al. 1998) has been used to block the increase in [Ca^2+^]_i_, but its recent application is uncommon. Briefly, both agents ultimately affect intracellular calcium levels. Given the widespread use of EGTA, we utilized it in the current study. Nevertheless, considering the importance of [Ca^2+^]_i_ regulation in therapeutic drug development, it is crucial to explore alternative approaches. A multifaceted approach is necessary to effectively target and modulate [Ca^2+^]_i_ levels, including exploring novel agents or techniques that can directly influence [Ca^2+^]_i_ dynamics. By doing so, we can gain deeper insights into calcium-related cellular processes and potentially develop more effective therapeutic strategies for treating calcium dysregulation-related diseases.

Calcium signaling and its network of interactions are involved in mediating many cellular physiological functions. Studies have shown that the agonist-induced Ca^2+^ level is critical for PLT activation in hemostasis and thrombosis(Varga-Szabo et al. 2009). Increased procoagulant activity of PLTs can be initiated by integrin αIIbβ3 (GPIIb/IIIa)/Gα13-mediated co-stimulation of outward-inward signaling and GPVI signaling, leading to intracellular Ca^2+^ release above a threshold(Kaiser et al. 2022). In a study on procoagulant PLTs, it was mentioned that only the procoagulant PLTs showed high cytoplasmic Ca^2+^ level as detected by fluorescent probes(Abbasian et al. 2020). Although the crucial significance of calcium ions in platelets and thrombosis is well-established, the potential perils associated with their interaction with TiO_2_NPs, particularly pertaining to calcium involvement, have remained relatively unexplored. This article endeavors to fill this gap in knowledge and enhance our understanding of this intricate relationship by demonstrating that exposure to TiO_2_NPs elevates intracellular Ca^2+^ levels, which are vital for triggering platelet activation, aggregation, and procoagulant activity, ultimately culminating in arterial thrombosis in mice. Thus, it is noteworthy that our study may provide new clues on the preventive and therapeutic strategies of AT caused by TiO_2_NP treatment.

## Conclusions

Our study reveals a pro-thrombotic effect of TiO_2_NPs by pro-coagulant activity and activation/aggregation of PLTs. Mechanistically, TiO_2_NPs initiate PS exposure and MVs generation, ultimately leading to a heightened pro-coagulant activity. Simultaneously, these particles boost the expression of P-selectin and activate GPIIb/IIIa, thereby facilitating the activation/aggregation of platelets. This entire process involves an increase in intracellular Ca^2+^ levels, making it calcium-dependent. Most importantly, we demonstrated the relevance of our findings in vivo by showing that intravenous administration of TiO_2_NPs can increase arterial thrombosis in mouse carotid arteries, emphasizing the need for caution during medical applications of TiO_2_NPs.

## Data Availability

No datasets were generated or analysed during the current study
